# [2-(*sec*-Butyl­imino­meth­yl)quinoline]­chlorido(η^6^-1-isopropyl-4-methyl­benzene)­ruthenium(II) hexa­fluorido­phosphate

**DOI:** 10.1107/S1600536810033003

**Published:** 2010-08-21

**Authors:** José Luis Gárate-Morales, Simón Hernández-Ortega, Juan Manuel Fernández-G

**Affiliations:** a Instituto de Química, Universidad Nacional Autónoma de México, Circuito Exterior, Ciudad Universitaria, México 04510, Mexico

## Abstract

In the title compound, [RuCl(C_10_H_14_)(C_14_H_16_N_2_)]PF_6_, the aromatic ring of the isopropyl­methyl­benzene fragment shows an η^6^-arene coordination to the ruthenium atom. Its coordination sphere is completed by a chloride ligand and 2-(*sec*-butyl­imino­meth­yl)quinoline. The dihedral angle between the η^6^-arene ring and the quinoline Schiff base is 45.64 (9)°. The *sec*-butyl substituent and the PF_6_^−^ anion are disordered over two positions with ratios of 0.595 (11):0.405 (11) and 0.752 (8):0.248 (8), respectively.

## Related literature

For the synthesis of a ruthenium dimer, see: Bennet *et al.* (1982 ▶). For the synthesis of the Schiff base ligand and a Schiff base–ruthenium arene complex, see: Moreno *et al.* (2009 ▶); Brunner *et al.* (2003 ▶); Lalrempuia *et al.* (2003 ▶). For the catalytic applications of Schiff base–ruthenium arene complexes, see: Drozdzak *et al.* (2005 ▶); Opstal *et al.* (2003 ▶).
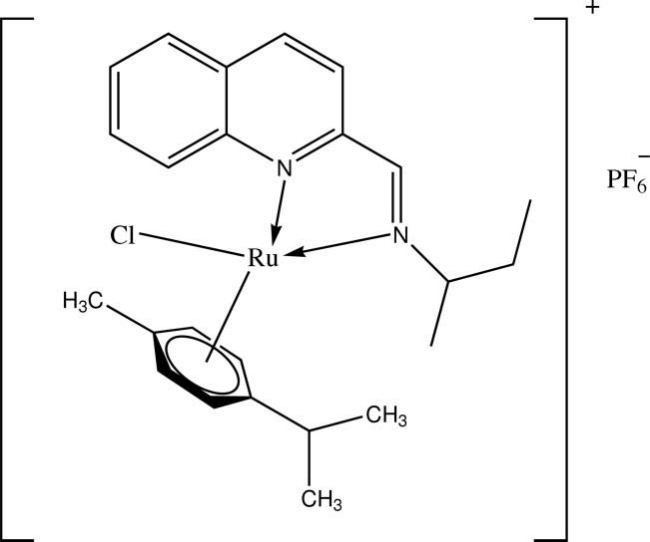


## Experimental

### 

#### Crystal data

[RuCl(C_10_H_14_)(C_14_H_16_N_2_)]PF_6_
*M* *_r_* = 627.99
Monoclinic, 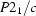

*a* = 10.4513 (6) Å
*b* = 15.3595 (9) Å
*c* = 16.578 (1) Å
β = 97.484 (1)°
*V* = 2638.5 (3) Å^3^
*Z* = 4
Mo *K*α radiation
μ = 0.81 mm^−1^
*T* = 298 K
0.35 × 0.31 × 0.08 mm

#### Data collection

Bruker SMART APEX CCD area-detector diffractometer
Absorption correction: multi-scan (*SHELXTL*; Sheldrick, 2008 ▶) *T*_min_ = 0.764, *T*_max_ = 0.938
21833 measured reflections
4822 independent reflections
3985 reflections with *I* > 2σ(*I*)
*R*_int_ = 0.048

#### Refinement

*R*[*F* ^2^ > 2σ(*F* ^2^)] = 0.038
*wR*(*F* ^2^) = 0.099
*S* = 1.03
4822 reflections
405 parameters
498 restraints
H-atom parameters constrained
Δρ_max_ = 0.71 e Å^−3^
Δρ_min_ = −0.40 e Å^−3^

### 

Data collection: *SMART* (Bruker, 1999 ▶); cell refinement: *SAINT* (Bruker, 1999 ▶); data reduction: *SAINT*; program(s) used to solve structure: *SHELXTL* (Sheldrick, 2008 ▶); program(s) used to refine structure: *SHELXTL*; molecular graphics: *SHELXTL*; software used to prepare material for publication: *SHELXTL*.

## Supplementary Material

Crystal structure: contains datablocks I, global. DOI: 10.1107/S1600536810033003/bt5317sup1.cif

Structure factors: contains datablocks I. DOI: 10.1107/S1600536810033003/bt5317Isup2.hkl

Additional supplementary materials:  crystallographic information; 3D view; checkCIF report

## Figures and Tables

**Fig. 1.**
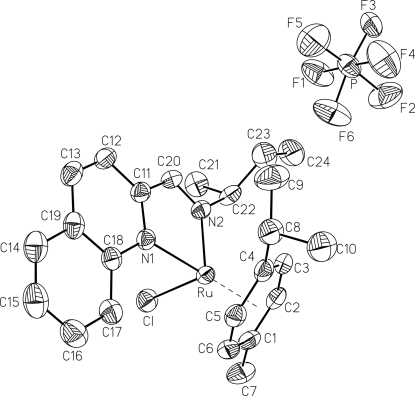
The molecular structure with numbering scheme. Displacement ellipsoids are drawn at the 40% probability level. The H atom bonded to C atom and disordered atoms were omitted for clarity.

## supplementary crystallographic information

### Comment

Schiff bases are found among the most convenient and attractive ligands for ruthenium complexes (Bennet *et al.* 1982), by steric and electronic effects around the Ru core, the donor atoms, N and O, of the chelated Schiff bases exert two opposite electronic effects (Brunner *et al.*, 2003; Lalrempuia  *et al.* 2003; Moreno *et al.* 2009]. As result of their particular structure, these ruthenium complexes display an enhanced activity and selectivity in a multitude of organic transformations [Drozdzak *et al.* 2005 & Opstal *et al.* 2003]. Due to importance of Schiff base ruthenium-arene complexes show as catalysts, we describe the synthesis and structural analysis of a new ruthenium Schiff base complex using an iminomethylquinoline as ligand.

The molecular structure of (I) with the numbering scheme is shown in Fig. 1. Selected bond distances, bond angles and torsion angles are shown in table 1. The structure exhibits an η6 - arene coordination of the isopropylmethylbenzene bonded to the ruthenium mononuclear structure, completing the coordinations sphere a chloride atom and 2-*N*-*sec*-butyliminomethylquinoline Schiff base are found. While the structure of classical Schiff bases have a fragment (O—C=C—C=N) and form a six member chelate ring with the metal atom as sixth member of the chelate, in (I) the Schiff base fragment (N1—C11—C20—N2) is forming a five member chelate ring with the ruthenium atom as fifth member of the chelate. The η6 - arene coordination and Schiff base fragment are not cooplanar and are forming a dihedral angle of 52.94°(9)°. The quinoline ring is sliglty deviated of planarity [0.044 (3) Å], while N1—C11—C20—N2 is planar [0.040 (2) Å]

### Experimental

Schiff base ligand. To a solution of 2-quinolincarboxaldehyde (1.0 g) in 150 ml of ethanol was added 0.64 ml (0.47 g) of *sec*-butylamine. The mixture was heated at 60 °C for 5 h, then concentrated until all the solvent was distilled. The residual deep yellow oil was then vacuum distilled obtaining 1.32 g of a light yellow liquid which was characterized by ^1^H NMR and IR spectroscopy.

Ruthenium derivative. To a solution of 0.9 g of di-*m*-Cl-bis([Cl(η6–1-isopropyl-4-methylbenzene)Ru(II)] in 40 ml of anhydrous methanol under nitrogen was added 0.0636 g of the 2-[*N*-(*sec*-butyliminomethyl)]quinoline followed of 0.110 g of potassium hexafluorophosphate the reaction mixture was stirred for 3 h. The color of the reaction mixture changed from initially orange to deep red at the end and by solvent concentration a precipitate was formed which was separated and dissolved in dichloromethane. The obtained solution was filtered through celite, concentrated and stored in a freezer at -30 °C for 48 h obtaining 0.75 g of a product as red crystals. This product (I) was characterized by IR spectroscopy and mass spectrometry (FAB+). Good quality crystals suitable for the X-ray diffraction study, were obtained by slow layer diffusion of methanol into a saturated dichloromethane solution of compound (I) at room temperature.

### Refinement

H   atoms were placed in geometrically idealized positions [0.98 Å (for CH), 0.97 Å (for CH_2_), 0.96 Å (for CH_3_) and 0.93Å (for CH aromatic)] and refined using the riding model with *U*_iso_(H) = 1.2 *U*_eq_(C) or 1.5 *U*_eq_C(methyl). The *sec*-butyl substituent and hexafluorophosphate anion are disordered and were refined anistropically in two positions with site occupation factors of 0.595 (11)/0.405 (11) and 0.752 (8)/0.248 (8), respectively.

### Crystal data

**Table:**

[RuCl(C_10_H_14_)(C_14_H_16_N_2_)]PF_6_	*F*(000) = 1272
*M**_r_* = 627.99	*D*_x_ = 1.581 Mg m^−^^3^
Monoclinic, *P*2_1_/*c*	Mo *K*α radiation, λ = 0.71073 Å
Hall symbol: -P 2ybc	Cell parameters from 6413 reflections
*a* = 10.4513 (6) Å	θ = 2.5–32.4°
*b* = 15.3595 (9) Å	µ = 0.81 mm^−^^1^
*c* = 16.578 (1) Å	*T* = 298 K
β = 97.484 (1)°	Prism, red
*V* = 2638.5 (3) Å^3^	0.35 × 0.31 × 0.08 mm
*Z* = 4	

### Data collection

**Table:**

Bruker SMART APEX CCD area-detector diffractometer	4822 independent reflections
Radiation source: fine-focus sealed tube	3985 reflections with *I* > 2σ(*I*)
graphite	*R*_int_ = 0.048
Detector resolution: 0.83 pixels mm^-1^	θ_max_ = 25.4°, θ_min_ = 1.8°
ω scans	*h* = −12→12
Absorption correction: multi-scan (*SHELXTL*; Sheldrick, 2008)	*k* = −18→18
*T*_min_ = 0.764, *T*_max_ = 0.938	*l* = −19→19
21833 measured reflections	

### Refinement

**Table:**

Refinement on *F*^2^	Primary atom site location: structure-invariant direct methods
Least-squares matrix: full	Secondary atom site location: difference Fourier map
*R*[*F*^2^ > 2σ(*F*^2^)] = 0.038	Hydrogen site location: inferred from neighbouring sites
*wR*(*F*^2^) = 0.099	H-atom parameters constrained
*S* = 1.03	*w* = 1/[σ^2^(*F*_o_^2^) + (0.062*P*)^2^] where *P* = (*F*_o_^2^ + 2*F*_c_^2^)/3
4822 reflections	(Δ/σ)_max_ = 0.016
405 parameters	Δρ_max_ = 0.71 e Å^−^^3^
498 restraints	Δρ_min_ = −0.40 e Å^−^^3^

### Special details

**Table:**

Geometry. All e.s.d.'s (except the e.s.d. in the dihedral angle between two l.s. planes) are estimated using the full covariance matrix. The cell e.s.d.'s are taken into account individually in the estimation of e.s.d.'s in distances, angles and torsion angles; correlations between e.s.d.'s in cell parameters are only used when they are defined by crystal symmetry. An approximate (isotropic) treatment of cell e.s.d.'s is used for estimating e.s.d.'s involving l.s. planes.
Refinement. Refinement of *F*^2^ against ALL reflections. The weighted *R*-factor *wR* and goodness of fit *S* are based on *F*^2^, conventional *R*-factors *R* are based on *F*, with *F* set to zero for negative *F*^2^. The threshold expression of *F*^2^ > σ(*F*^2^) is used only for calculating *R*-factors(gt) *etc*. and is not relevant to the choice of reflections for refinement. *R*-factors based on *F*^2^ are statistically about twice as large as those based on *F*, and *R*- factors based on ALL data will be even larger.

### Fractional atomic coordinates and isotropic or equivalent isotropic displacement parameters (Å^2^)

**Table:**

	*x*	*y*	*z*	*U*_iso_*/*U*_eq_	Occ. (<1)
Ru	0.84887 (3)	−0.039403 (18)	0.232792 (16)	0.03610 (13)	
Cl	0.76723 (11)	−0.13951 (7)	0.32372 (6)	0.0547 (3)	
N1	0.9567 (3)	0.01445 (19)	0.33846 (17)	0.0391 (7)	
C1	0.8165 (4)	−0.1398 (2)	0.1343 (2)	0.0477 (9)	
C2	0.7535 (4)	−0.0629 (3)	0.1085 (2)	0.0473 (10)	
H2	0.6662	−0.0645	0.0885	0.057*	
C3	0.8200 (4)	0.0176 (3)	0.1121 (2)	0.0458 (9)	
H3	0.7748	0.0680	0.0953	0.055*	
C4	0.9518 (4)	0.0233 (3)	0.1402 (2)	0.0449 (9)	
C5	1.0150 (4)	−0.0553 (2)	0.1654 (2)	0.0442 (9)	
H5	1.1028	−0.0539	0.1841	0.053*	
C6	0.9506 (4)	−0.1343 (3)	0.1633 (2)	0.0468 (9)	
H6	0.9957	−0.1845	0.1810	0.056*	
C7	0.7454 (4)	−0.2243 (3)	0.1338 (3)	0.0688 (13)	
H7A	0.6621	−0.2146	0.1506	0.103*	
H7B	0.7935	−0.2642	0.1706	0.103*	
H7C	0.7350	−0.2482	0.0799	0.103*	
C8	1.0292 (4)	0.1076 (3)	0.1447 (3)	0.0579 (11)	
H8	1.1027	0.1002	0.1873	0.069*	
C9	0.9586 (6)	0.1858 (3)	0.1652 (3)	0.0872 (17)	
H9A	0.8861	0.1952	0.1243	0.131*	
H9B	1.0149	0.2354	0.1676	0.131*	
H9C	0.9288	0.1778	0.2171	0.131*	
C10	1.0837 (5)	0.1192 (3)	0.0638 (3)	0.0756 (14)	
H10A	1.0140	0.1219	0.0201	0.113*	
H10B	1.1385	0.0707	0.0554	0.113*	
H10C	1.1328	0.1721	0.0653	0.113*	
C11	0.8981 (4)	0.0824 (2)	0.3693 (2)	0.0417 (9)	
C12	0.9552 (4)	0.1328 (3)	0.4349 (2)	0.0490 (10)	
H12	0.9110	0.1796	0.4536	0.059*	
C13	1.0758 (4)	0.1125 (3)	0.4707 (2)	0.0518 (10)	
H13	1.1160	0.1463	0.5131	0.062*	
C14	1.2623 (4)	0.0126 (3)	0.4809 (3)	0.0605 (12)	
H14	1.3056	0.0458	0.5227	0.073*	
C15	1.3161 (5)	−0.0605 (4)	0.4570 (3)	0.0713 (14)	
H15	1.3969	−0.0775	0.4823	0.086*	
C16	1.2523 (4)	−0.1116 (3)	0.3943 (3)	0.0666 (12)	
H16	1.2902	−0.1629	0.3793	0.080*	
C17	1.1349 (4)	−0.0869 (3)	0.3551 (2)	0.0533 (10)	
H17	1.0938	−0.1211	0.3132	0.064*	
C18	1.0763 (4)	−0.0103 (3)	0.3775 (2)	0.0415 (9)	
C19	1.1397 (4)	0.0394 (3)	0.4428 (2)	0.0481 (10)	
C20	0.7683 (4)	0.0977 (2)	0.3317 (2)	0.0450 (9)	
H20	0.7184	0.1414	0.3507	0.054*	
N2	0.7229 (3)	0.05014 (19)	0.27189 (18)	0.0416 (7)	
C21	0.5006 (14)	0.0530 (11)	0.2997 (13)	0.066 (4)	0.595 (11)
H21A	0.5109	−0.0056	0.3195	0.099*	0.595 (11)
H21B	0.4122	0.0626	0.2777	0.099*	0.595 (11)
H21C	0.5245	0.0929	0.3437	0.099*	0.595 (11)
C22	0.5868 (9)	0.0677 (14)	0.2335 (12)	0.055 (4)	0.595 (11)
H22	0.5643	0.0238	0.1911	0.066*	0.595 (11)
C23	0.5702 (15)	0.1587 (16)	0.1931 (12)	0.076 (5)	0.595 (11)
H23A	0.5803	0.2044	0.2338	0.092*	0.595 (11)
H23B	0.6329	0.1675	0.1556	0.092*	0.595 (11)
C24	0.4327 (9)	0.1578 (7)	0.1479 (6)	0.104 (4)	0.595 (11)
H24A	0.4139	0.2133	0.1224	0.156*	0.595 (11)
H24B	0.3726	0.1467	0.1858	0.156*	0.595 (11)
H24C	0.4254	0.1130	0.1072	0.156*	0.595 (11)
C21A	0.558 (2)	0.148 (2)	0.2066 (16)	0.078 (8)	0.405 (11)
H21D	0.6157	0.1617	0.1677	0.117*	0.405 (11)
H21E	0.5731	0.1875	0.2517	0.117*	0.405 (11)
H21F	0.4707	0.1531	0.1814	0.117*	0.405 (11)
C22A	0.5835 (12)	0.0541 (18)	0.2372 (16)	0.049 (6)	0.405 (11)
H22A	0.5717	0.0157	0.1896	0.059*	0.405 (11)
C23A	0.4888 (18)	0.0237 (18)	0.295 (2)	0.086 (8)	0.405 (11)
H23C	0.5135	0.0462	0.3497	0.103*	0.405 (11)
H23D	0.4850	−0.0393	0.2975	0.103*	0.405 (11)
C24A	0.3582 (9)	0.0622 (10)	0.2571 (9)	0.089 (5)	0.405 (11)
H24D	0.2934	0.0498	0.2915	0.134*	0.405 (11)
H24E	0.3336	0.0366	0.2045	0.134*	0.405 (11)
H24F	0.3663	0.1241	0.2514	0.134*	0.405 (11)
P	0.33938 (11)	0.84242 (8)	0.08714 (8)	0.0627 (3)	
F1	0.2464 (7)	0.8093 (5)	0.1477 (5)	0.114 (2)	0.752 (8)
F2	0.2477 (7)	0.8018 (4)	0.0124 (4)	0.136 (2)	0.752 (8)
F3	0.2633 (6)	0.9293 (3)	0.0735 (4)	0.1032 (18)	0.752 (8)
F4	0.4317 (7)	0.8723 (5)	0.0237 (4)	0.127 (2)	0.752 (8)
F5	0.4400 (7)	0.8762 (4)	0.1569 (4)	0.126 (2)	0.752 (8)
F6	0.4127 (5)	0.7502 (3)	0.0936 (4)	0.0839 (13)	0.752 (8)
F1A	0.4061 (18)	0.8896 (12)	0.0215 (10)	0.112 (4)	0.248 (8)
F2A	0.4669 (11)	0.8445 (12)	0.1499 (10)	0.100 (4)	0.248 (8)
F3A	0.3684 (18)	0.7491 (6)	0.0610 (12)	0.105 (4)	0.248 (8)
F4A	0.2690 (18)	0.8007 (12)	0.1588 (9)	0.081 (4)	0.248 (8)
F5A	0.2037 (10)	0.8473 (12)	0.0352 (9)	0.104 (4)	0.248 (8)
F6A	0.3059 (14)	0.9359 (6)	0.1252 (11)	0.0839 (13)	0.248 (8)

### Atomic displacement parameters (Å^2^)

**Table:**

	*U*^11^	*U*^22^	*U*^33^	*U*^12^	*U*^13^	*U*^23^
Ru	0.04056 (19)	0.03523 (19)	0.03323 (18)	0.00114 (13)	0.00754 (12)	−0.00198 (12)
Cl	0.0679 (7)	0.0506 (6)	0.0478 (6)	−0.0095 (5)	0.0151 (5)	0.0042 (4)
N1	0.0466 (18)	0.0392 (17)	0.0319 (16)	−0.0035 (14)	0.0070 (14)	0.0022 (13)
C1	0.057 (2)	0.044 (2)	0.045 (2)	−0.0017 (19)	0.0183 (19)	−0.0135 (17)
C2	0.047 (2)	0.060 (3)	0.034 (2)	0.0028 (19)	0.0045 (17)	−0.0132 (18)
C3	0.061 (3)	0.046 (2)	0.0298 (19)	0.0066 (19)	0.0066 (17)	−0.0003 (16)
C4	0.055 (2)	0.050 (2)	0.0320 (19)	−0.0026 (19)	0.0150 (17)	−0.0018 (17)
C5	0.042 (2)	0.054 (2)	0.039 (2)	0.0015 (18)	0.0125 (17)	−0.0023 (17)
C6	0.052 (2)	0.044 (2)	0.046 (2)	0.0097 (19)	0.0145 (18)	−0.0035 (17)
C7	0.077 (3)	0.054 (3)	0.078 (3)	−0.013 (2)	0.019 (3)	−0.021 (2)
C8	0.068 (3)	0.054 (3)	0.054 (3)	−0.010 (2)	0.021 (2)	0.001 (2)
C9	0.112 (4)	0.058 (3)	0.099 (4)	−0.017 (3)	0.044 (4)	−0.016 (3)
C10	0.094 (4)	0.067 (3)	0.073 (3)	−0.012 (3)	0.040 (3)	0.005 (3)
C11	0.048 (2)	0.041 (2)	0.037 (2)	−0.0031 (18)	0.0114 (17)	0.0000 (16)
C12	0.062 (3)	0.045 (2)	0.042 (2)	−0.004 (2)	0.0100 (19)	−0.0067 (17)
C13	0.065 (3)	0.052 (2)	0.038 (2)	−0.017 (2)	0.006 (2)	−0.0041 (18)
C14	0.053 (3)	0.076 (3)	0.050 (3)	−0.009 (2)	−0.001 (2)	0.005 (2)
C15	0.052 (3)	0.095 (4)	0.064 (3)	0.002 (3)	−0.003 (2)	0.014 (3)
C16	0.060 (3)	0.068 (3)	0.072 (3)	0.011 (2)	0.007 (2)	0.008 (3)
C17	0.052 (2)	0.055 (3)	0.052 (2)	0.003 (2)	0.003 (2)	0.004 (2)
C18	0.042 (2)	0.046 (2)	0.038 (2)	−0.0035 (17)	0.0096 (17)	0.0070 (17)
C19	0.048 (2)	0.057 (3)	0.039 (2)	−0.0126 (19)	0.0052 (18)	0.0081 (18)
C20	0.054 (2)	0.039 (2)	0.043 (2)	0.0060 (18)	0.0097 (18)	−0.0070 (17)
N2	0.0445 (18)	0.0437 (18)	0.0376 (17)	0.0053 (14)	0.0091 (14)	−0.0045 (14)
C21	0.040 (5)	0.082 (10)	0.075 (8)	−0.014 (5)	−0.001 (5)	−0.001 (7)
C22	0.050 (8)	0.064 (9)	0.051 (8)	0.001 (6)	0.010 (6)	−0.021 (7)
C23	0.068 (8)	0.093 (12)	0.072 (8)	0.028 (7)	0.023 (7)	0.009 (7)
C24	0.089 (7)	0.109 (8)	0.104 (8)	0.036 (6)	−0.020 (6)	0.004 (6)
C21A	0.080 (15)	0.066 (13)	0.076 (14)	0.020 (11)	−0.033 (12)	0.005 (11)
C22A	0.046 (11)	0.047 (9)	0.052 (11)	0.020 (8)	−0.003 (10)	0.001 (8)
C23A	0.059 (11)	0.119 (19)	0.085 (12)	−0.017 (10)	0.032 (9)	−0.035 (13)
C24A	0.050 (7)	0.124 (13)	0.092 (10)	−0.018 (7)	0.004 (7)	−0.027 (9)
P	0.0546 (7)	0.0548 (7)	0.0768 (8)	−0.0068 (6)	0.0009 (6)	0.0149 (6)
F1	0.097 (4)	0.097 (4)	0.161 (5)	0.034 (3)	0.066 (4)	0.056 (3)
F2	0.141 (4)	0.114 (4)	0.132 (4)	−0.009 (3)	−0.057 (3)	−0.015 (3)
F3	0.132 (4)	0.078 (2)	0.104 (4)	0.038 (3)	0.030 (3)	0.036 (3)
F4	0.125 (4)	0.117 (4)	0.151 (4)	0.006 (3)	0.063 (4)	0.058 (3)
F5	0.122 (4)	0.107 (4)	0.135 (4)	−0.008 (3)	−0.031 (3)	−0.042 (3)
F6	0.083 (3)	0.0628 (19)	0.108 (4)	0.0081 (19)	0.023 (2)	0.0114 (19)
F1A	0.101 (8)	0.113 (9)	0.135 (8)	0.014 (8)	0.058 (7)	0.048 (7)
F2A	0.052 (5)	0.130 (11)	0.113 (8)	0.017 (5)	−0.008 (5)	−0.025 (8)
F3A	0.141 (10)	0.074 (5)	0.104 (10)	−0.006 (6)	0.029 (7)	−0.018 (6)
F4A	0.081 (7)	0.081 (7)	0.084 (6)	0.018 (7)	0.018 (5)	0.036 (6)
F5A	0.082 (5)	0.124 (9)	0.095 (7)	−0.021 (6)	−0.028 (6)	0.038 (6)
F6A	0.083 (3)	0.0628 (19)	0.108 (4)	0.0081 (19)	0.023 (2)	0.0114 (19)

### Geometric parameters (Å, °)

**Table:**

Ru—N2	2.066 (3)	C16—C17	1.366 (6)
Ru—N1	2.123 (3)	C16—H16	0.9300
Ru—C3	2.169 (4)	C17—C18	1.399 (6)
Ru—C5	2.197 (4)	C17—H17	0.9300
Ru—C2	2.199 (4)	C18—C19	1.417 (5)
Ru—C4	2.207 (4)	C20—N2	1.272 (4)
Ru—C6	2.215 (4)	C20—H20	0.9300
Ru—C1	2.239 (4)	N2—C22A	1.496 (9)
Ru—Cl	2.3875 (10)	N2—C22	1.504 (8)
N1—C11	1.345 (5)	C21—C22	1.525 (12)
N1—C18	1.383 (5)	C21—H21A	0.9600
C1—C2	1.391 (5)	C21—H21B	0.9600
C1—C6	1.424 (6)	C21—H21C	0.9600
C1—C7	1.495 (5)	C22—C23	1.550 (13)
C2—C3	1.416 (5)	C22—H22	0.9800
C2—H2	0.9300	C23—C24	1.532 (16)
C3—C4	1.398 (6)	C23—H23A	0.9700
C3—H3	0.9300	C23—H23B	0.9700
C4—C5	1.412 (5)	C24—H24A	0.9600
C4—C8	1.524 (5)	C24—H24B	0.9600
C5—C6	1.386 (5)	C24—H24C	0.9600
C5—H5	0.9300	C21A—C22A	1.540 (16)
C6—H6	0.9300	C21A—H21D	0.9600
C7—H7A	0.9600	C21A—H21E	0.9600
C7—H7B	0.9600	C21A—H21F	0.9600
C7—H7C	0.9600	C22A—C23A	1.539 (16)
C8—C9	1.472 (6)	C22A—H22A	0.9800
C8—C10	1.534 (5)	C23A—C24A	1.545 (19)
C8—H8	0.9800	C23A—H23C	0.9700
C9—H9A	0.9600	C23A—H23D	0.9700
C9—H9B	0.9600	C24A—H24D	0.9600
C9—H9C	0.9600	C24A—H24E	0.9600
C10—H10A	0.9600	C24A—H24F	0.9600
C10—H10B	0.9600	P—F3A	1.539 (7)
C10—H10C	0.9600	P—F1A	1.547 (7)
C11—C12	1.402 (5)	P—F5	1.548 (4)
C11—C20	1.437 (5)	P—F3	1.554 (4)
C12—C13	1.358 (5)	P—F5A	1.562 (7)
C12—H12	0.9300	P—F1	1.570 (4)
C13—C19	1.414 (6)	P—F2A	1.580 (7)
C13—H13	0.9300	P—F4	1.585 (4)
C14—C15	1.339 (7)	P—F2	1.593 (4)
C14—C19	1.413 (6)	P—F6	1.608 (4)
C14—H14	0.9300	P—F4A	1.609 (7)
C15—C16	1.400 (6)	P—F6A	1.625 (7)
C15—H15	0.9300		
			
N2—Ru—N1	76.90 (12)	C17—C18—C19	118.6 (4)
N2—Ru—C3	90.17 (14)	C14—C19—C13	121.6 (4)
N1—Ru—C3	126.81 (13)	C14—C19—C18	119.4 (4)
N2—Ru—C5	144.25 (13)	C13—C19—C18	118.9 (4)
N1—Ru—C5	95.33 (13)	N2—C20—C11	118.7 (3)
C3—Ru—C5	66.42 (15)	N2—C20—H20	120.7
N2—Ru—C2	100.02 (13)	C11—C20—H20	120.7
N1—Ru—C2	164.63 (13)	C20—N2—C22A	121.4 (9)
C3—Ru—C2	37.82 (14)	C20—N2—C22	117.6 (6)
C5—Ru—C2	78.28 (14)	C20—N2—Ru	116.0 (3)
N2—Ru—C4	108.42 (13)	C22A—N2—Ru	122.3 (8)
N1—Ru—C4	98.59 (13)	C22—N2—Ru	126.3 (6)
C3—Ru—C4	37.26 (15)	C22—C21—H21A	109.5
C5—Ru—C4	37.42 (14)	C22—C21—H21B	109.5
C2—Ru—C4	67.81 (15)	H21A—C21—H21B	109.5
N2—Ru—C6	166.04 (14)	C22—C21—H21C	109.5
N1—Ru—C6	116.52 (13)	H21A—C21—H21C	109.5
C3—Ru—C6	78.70 (14)	H21B—C21—H21C	109.5
C5—Ru—C6	36.63 (13)	N2—C22—C21	106.5 (12)
C2—Ru—C6	66.02 (15)	N2—C22—C23	113.0 (13)
C4—Ru—C6	67.34 (14)	C21—C22—C23	113.6 (7)
N2—Ru—C1	130.19 (14)	N2—C22—H22	107.8
N1—Ru—C1	152.05 (14)	C21—C22—H22	107.8
C3—Ru—C1	67.37 (14)	C23—C22—H22	107.8
C5—Ru—C1	66.86 (14)	C24—C23—C22	104.4 (12)
C2—Ru—C1	36.52 (14)	C24—C23—H23A	110.9
C4—Ru—C1	80.45 (14)	C22—C23—H23A	110.9
C6—Ru—C1	37.27 (14)	C24—C23—H23B	110.9
N2—Ru—Cl	86.32 (9)	C22—C23—H23B	110.9
N1—Ru—Cl	85.70 (8)	H23A—C23—H23B	108.9
C3—Ru—Cl	145.50 (11)	C23—C24—H24A	109.5
C5—Ru—Cl	128.37 (10)	C23—C24—H24B	109.5
C2—Ru—Cl	109.28 (11)	H24A—C24—H24B	109.5
C4—Ru—Cl	165.21 (11)	C23—C24—H24C	109.5
C6—Ru—Cl	98.05 (11)	H24A—C24—H24C	109.5
C1—Ru—Cl	89.12 (10)	H24B—C24—H24C	109.5
C11—N1—C18	117.6 (3)	C22A—C21A—H21D	109.5
C11—N1—Ru	113.4 (2)	C22A—C21A—H21E	109.5
C18—N1—Ru	129.0 (2)	H21D—C21A—H21E	109.5
C2—C1—C6	117.4 (4)	C22A—C21A—H21F	109.5
C2—C1—C7	121.3 (4)	H21D—C21A—H21F	109.5
C6—C1—C7	121.3 (4)	H21E—C21A—H21F	109.5
C2—C1—Ru	70.2 (2)	N2—C22A—C23A	114.9 (16)
C6—C1—Ru	70.4 (2)	N2—C22A—C21A	106.3 (16)
C7—C1—Ru	129.0 (3)	C23A—C22A—C21A	113.2 (9)
C1—C2—C3	121.2 (4)	N2—C22A—H22A	107.4
C1—C2—Ru	73.3 (2)	C23A—C22A—H22A	107.4
C3—C2—Ru	69.9 (2)	C21A—C22A—H22A	107.4
C1—C2—H2	119.4	C22A—C23A—C24A	103.5 (13)
C3—C2—H2	119.4	C22A—C23A—H23C	111.1
Ru—C2—H2	130.0	C24A—C23A—H23C	111.1
C4—C3—C2	121.7 (4)	C22A—C23A—H23D	111.1
C4—C3—Ru	72.9 (2)	C24A—C23A—H23D	111.1
C2—C3—Ru	72.3 (2)	H23C—C23A—H23D	109.0
C4—C3—H3	119.1	C23A—C24A—H24D	109.5
C2—C3—H3	119.1	C23A—C24A—H24E	109.5
Ru—C3—H3	128.0	H24D—C24A—H24E	109.5
C3—C4—C5	116.6 (4)	C23A—C24A—H24F	109.5
C3—C4—C8	124.3 (4)	H24D—C24A—H24F	109.5
C5—C4—C8	119.1 (4)	H24E—C24A—H24F	109.5
C3—C4—Ru	69.9 (2)	F3A—P—F1A	96.6 (7)
C5—C4—Ru	70.9 (2)	F3A—P—F5	112.6 (7)
C8—C4—Ru	129.8 (3)	F1A—P—F5	92.5 (10)
C6—C5—C4	122.3 (4)	F3A—P—F3	150.9 (7)
C6—C5—Ru	72.4 (2)	F1A—P—F3	76.4 (8)
C4—C5—Ru	71.7 (2)	F5—P—F3	96.1 (3)
C6—C5—H5	118.9	F3A—P—F5A	95.0 (7)
C4—C5—H5	118.9	F1A—P—F5A	92.9 (7)
Ru—C5—H5	129.7	F5—P—F5A	151.1 (7)
C5—C6—C1	120.9 (4)	F3—P—F5A	57.9 (6)
C5—C6—Ru	71.0 (2)	F3A—P—F1	92.3 (9)
C1—C6—Ru	72.3 (2)	F1A—P—F1	167.1 (8)
C5—C6—H6	119.6	F5—P—F1	92.8 (4)
C1—C6—H6	119.6	F3—P—F1	91.4 (3)
Ru—C6—H6	129.7	F5A—P—F1	77.0 (7)
C1—C7—H7A	109.5	F3A—P—F2A	91.4 (7)
C1—C7—H7B	109.5	F1A—P—F2A	92.0 (8)
H7A—C7—H7B	109.5	F3—P—F2A	116.7 (6)
C1—C7—H7C	109.5	F5A—P—F2A	171.4 (7)
H7A—C7—H7C	109.5	F1—P—F2A	97.2 (9)
H7B—C7—H7C	109.5	F3A—P—F4	85.6 (8)
C9—C8—C4	115.2 (4)	F5—P—F4	89.1 (4)
C9—C8—C10	111.2 (4)	F3—P—F4	89.9 (3)
C4—C8—C10	108.1 (3)	F5A—P—F4	102.0 (7)
C9—C8—H8	107.3	F1—P—F4	177.6 (4)
C4—C8—H8	107.3	F2A—P—F4	84.1 (9)
C10—C8—H8	107.3	F3A—P—F2	62.3 (7)
C8—C9—H9A	109.5	F1A—P—F2	85.2 (10)
C8—C9—H9B	109.5	F5—P—F2	174.0 (3)
H9A—C9—H9B	109.5	F3—P—F2	88.8 (3)
C8—C9—H9C	109.5	F1—P—F2	90.6 (4)
H9A—C9—H9C	109.5	F2A—P—F2	153.0 (6)
H9B—C9—H9C	109.5	F4—P—F2	87.4 (4)
C8—C10—H10A	109.5	F1A—P—F6	101.8 (8)
C8—C10—H10B	109.5	F5—P—F6	88.5 (3)
H10A—C10—H10B	109.5	F3—P—F6	175.1 (3)
C8—C10—H10C	109.5	F5A—P—F6	118.0 (7)
H10A—C10—H10C	109.5	F1—P—F6	90.1 (3)
H10B—C10—H10C	109.5	F2A—P—F6	67.7 (6)
N1—C11—C12	123.6 (4)	F4—P—F6	88.5 (3)
N1—C11—C20	114.4 (3)	F2—P—F6	86.5 (3)
C12—C11—C20	121.9 (4)	F3A—P—F4A	87.9 (7)
C13—C12—C11	119.3 (4)	F1A—P—F4A	175.5 (9)
C13—C12—H12	120.4	F5—P—F4A	85.0 (9)
C11—C12—H12	120.4	F3—P—F4A	100.1 (7)
C12—C13—C19	119.4 (4)	F5A—P—F4A	87.5 (7)
C12—C13—H13	120.3	F2A—P—F4A	87.1 (7)
C19—C13—H13	120.3	F4—P—F4A	168.9 (8)
C15—C14—C19	120.2 (4)	F2—P—F4A	97.6 (8)
C15—C14—H14	119.9	F6—P—F4A	82.0 (8)
C19—C14—H14	119.9	F3A—P—F6A	173.1 (7)
C14—C15—C16	120.8 (4)	F1A—P—F6A	89.9 (7)
C14—C15—H15	119.6	F5—P—F6A	64.7 (6)
C16—C15—H15	119.6	F3—P—F6A	33.5 (5)
C17—C16—C15	120.6 (5)	F5A—P—F6A	86.9 (6)
C17—C16—H16	119.7	F1—P—F6A	81.7 (6)
C15—C16—H16	119.7	F2A—P—F6A	86.1 (6)
C16—C17—C18	120.3 (4)	F4—P—F6A	100.5 (6)
C16—C17—H17	119.8	F2—P—F6A	120.7 (6)
C18—C17—H17	119.8	F6—P—F6A	151.4 (6)
N1—C18—C17	120.5 (3)	F4A—P—F6A	85.6 (7)
N1—C18—C19	120.9 (4)		
			
N2—Ru—N1—C11	−7.3 (2)	C2—Ru—C5—C6	−65.4 (2)
C3—Ru—N1—C11	72.9 (3)	C4—Ru—C5—C6	−134.1 (4)
C5—Ru—N1—C11	137.3 (3)	C1—Ru—C5—C6	−28.9 (2)
C2—Ru—N1—C11	72.8 (6)	Cl—Ru—C5—C6	40.0 (3)
C4—Ru—N1—C11	99.8 (3)	N2—Ru—C5—C4	−22.2 (4)
C6—Ru—N1—C11	168.7 (2)	N1—Ru—C5—C4	−97.2 (2)
C1—Ru—N1—C11	−174.4 (3)	C3—Ru—C5—C4	30.8 (2)
Cl—Ru—N1—C11	−94.5 (2)	C2—Ru—C5—C4	68.7 (2)
N2—Ru—N1—C18	173.5 (3)	C6—Ru—C5—C4	134.1 (4)
C3—Ru—N1—C18	−106.3 (3)	C1—Ru—C5—C4	105.2 (3)
C5—Ru—N1—C18	−41.9 (3)	Cl—Ru—C5—C4	174.09 (18)
C2—Ru—N1—C18	−106.4 (5)	C4—C5—C6—C1	0.7 (6)
C4—Ru—N1—C18	−79.5 (3)	Ru—C5—C6—C1	54.5 (3)
C6—Ru—N1—C18	−10.6 (3)	C4—C5—C6—Ru	−53.8 (3)
C1—Ru—N1—C18	6.4 (5)	C2—C1—C6—C5	0.0 (5)
Cl—Ru—N1—C18	86.3 (3)	C7—C1—C6—C5	−178.3 (4)
N2—Ru—C1—C2	40.7 (3)	Ru—C1—C6—C5	−53.9 (3)
N1—Ru—C1—C2	−155.8 (3)	C2—C1—C6—Ru	53.9 (3)
C3—Ru—C1—C2	−28.9 (2)	C7—C1—C6—Ru	−124.4 (4)
C5—Ru—C1—C2	−101.9 (3)	N2—Ru—C6—C5	103.2 (5)
C4—Ru—C1—C2	−65.4 (2)	N1—Ru—C6—C5	−60.2 (3)
C6—Ru—C1—C2	−130.3 (3)	C3—Ru—C6—C5	65.5 (2)
Cl—Ru—C1—C2	125.1 (2)	C2—Ru—C6—C5	103.0 (3)
N2—Ru—C1—C6	171.0 (2)	C4—Ru—C6—C5	28.2 (2)
N1—Ru—C1—C6	−25.4 (4)	C1—Ru—C6—C5	132.8 (4)
C3—Ru—C1—C6	101.4 (3)	Cl—Ru—C6—C5	−149.4 (2)
C5—Ru—C1—C6	28.4 (2)	N2—Ru—C6—C1	−29.7 (6)
C2—Ru—C1—C6	130.3 (3)	N1—Ru—C6—C1	167.0 (2)
C4—Ru—C1—C6	64.9 (2)	C3—Ru—C6—C1	−67.3 (2)
Cl—Ru—C1—C6	−104.6 (2)	C5—Ru—C6—C1	−132.8 (4)
N2—Ru—C1—C7	−74.1 (4)	C2—Ru—C6—C1	−29.8 (2)
N1—Ru—C1—C7	89.5 (5)	C4—Ru—C6—C1	−104.6 (3)
C3—Ru—C1—C7	−143.7 (4)	Cl—Ru—C6—C1	77.8 (2)
C5—Ru—C1—C7	143.4 (4)	C3—C4—C8—C9	34.9 (6)
C2—Ru—C1—C7	−114.8 (5)	C5—C4—C8—C9	−145.4 (4)
C4—Ru—C1—C7	179.8 (4)	Ru—C4—C8—C9	−56.7 (6)
C6—Ru—C1—C7	114.9 (5)	C3—C4—C8—C10	−90.2 (5)
Cl—Ru—C1—C7	10.4 (4)	C5—C4—C8—C10	89.5 (5)
C6—C1—C2—C3	−0.9 (5)	Ru—C4—C8—C10	178.2 (3)
C7—C1—C2—C3	177.4 (4)	C18—N1—C11—C12	4.6 (5)
Ru—C1—C2—C3	53.1 (3)	Ru—N1—C11—C12	−174.7 (3)
C6—C1—C2—Ru	−54.0 (3)	C18—N1—C11—C20	−172.9 (3)
C7—C1—C2—Ru	124.3 (4)	Ru—N1—C11—C20	7.8 (4)
N2—Ru—C2—C1	−149.6 (2)	N1—C11—C12—C13	−0.7 (6)
N1—Ru—C2—C1	133.4 (5)	C20—C11—C12—C13	176.6 (4)
C3—Ru—C2—C1	133.2 (3)	C11—C12—C13—C19	−1.9 (6)
C5—Ru—C2—C1	66.8 (2)	C19—C14—C15—C16	−0.2 (7)
C4—Ru—C2—C1	104.5 (3)	C14—C15—C16—C17	1.5 (7)
C6—Ru—C2—C1	30.4 (2)	C15—C16—C17—C18	−0.6 (7)
Cl—Ru—C2—C1	−60.0 (2)	C11—N1—C18—C17	171.8 (3)
N2—Ru—C2—C3	77.1 (2)	Ru—N1—C18—C17	−9.0 (5)
N1—Ru—C2—C3	0.2 (6)	C11—N1—C18—C19	−5.9 (5)
C5—Ru—C2—C3	−66.5 (2)	Ru—N1—C18—C19	173.3 (2)
C4—Ru—C2—C3	−28.8 (2)	C16—C17—C18—N1	−179.3 (4)
C6—Ru—C2—C3	−102.9 (3)	C16—C17—C18—C19	−1.5 (6)
C1—Ru—C2—C3	−133.2 (3)	C15—C14—C19—C13	175.1 (4)
Cl—Ru—C2—C3	166.7 (2)	C15—C14—C19—C18	−2.0 (6)
C1—C2—C3—C4	1.2 (6)	C12—C13—C19—C14	−176.4 (4)
Ru—C2—C3—C4	55.8 (3)	C12—C13—C19—C18	0.6 (6)
C1—C2—C3—Ru	−54.6 (3)	N1—C18—C19—C14	−179.5 (3)
N2—Ru—C3—C4	121.2 (2)	C17—C18—C19—C14	2.8 (5)
N1—Ru—C3—C4	47.5 (3)	N1—C18—C19—C13	3.4 (5)
C5—Ru—C3—C4	−31.0 (2)	C17—C18—C19—C13	−174.3 (4)
C2—Ru—C3—C4	−132.6 (3)	N1—C11—C20—N2	−3.2 (5)
C6—Ru—C3—C4	−67.3 (2)	C12—C11—C20—N2	179.2 (4)
C1—Ru—C3—C4	−104.5 (3)	C11—C20—N2—C22A	171.5 (15)
Cl—Ru—C3—C4	−155.06 (19)	C11—C20—N2—C22	−179.9 (12)
N2—Ru—C3—C2	−106.2 (2)	C11—C20—N2—Ru	−3.4 (5)
N1—Ru—C3—C2	−179.9 (2)	N1—Ru—N2—C20	5.7 (3)
C5—Ru—C3—C2	101.6 (2)	C3—Ru—N2—C20	−122.2 (3)
C4—Ru—C3—C2	132.6 (3)	C5—Ru—N2—C20	−75.1 (4)
C6—Ru—C3—C2	65.3 (2)	C2—Ru—N2—C20	−158.9 (3)
C1—Ru—C3—C2	28.0 (2)	C4—Ru—N2—C20	−89.1 (3)
Cl—Ru—C3—C2	−22.5 (3)	C6—Ru—N2—C20	−159.0 (5)
C2—C3—C4—C5	−0.5 (5)	C1—Ru—N2—C20	177.9 (3)
Ru—C3—C4—C5	55.1 (3)	Cl—Ru—N2—C20	92.1 (3)
C2—C3—C4—C8	179.3 (3)	N1—Ru—N2—C22A	−169.1 (15)
Ru—C3—C4—C8	−125.2 (4)	C3—Ru—N2—C22A	63.0 (15)
C2—C3—C4—Ru	−55.6 (3)	C5—Ru—N2—C22A	110.1 (15)
N2—Ru—C4—C3	−64.4 (2)	C2—Ru—N2—C22A	26.3 (15)
N1—Ru—C4—C3	−143.3 (2)	C4—Ru—N2—C22A	96.1 (15)
C5—Ru—C4—C3	129.1 (3)	C6—Ru—N2—C22A	26.2 (16)
C2—Ru—C4—C3	29.2 (2)	C1—Ru—N2—C22A	3.1 (15)
C6—Ru—C4—C3	101.4 (2)	Cl—Ru—N2—C22A	−82.7 (15)
C1—Ru—C4—C3	65.0 (2)	N1—Ru—N2—C22	−178.1 (13)
Cl—Ru—C4—C3	110.7 (4)	C3—Ru—N2—C22	53.9 (13)
N2—Ru—C4—C5	166.5 (2)	C5—Ru—N2—C22	101.0 (13)
N1—Ru—C4—C5	87.5 (2)	C2—Ru—N2—C22	17.2 (13)
C3—Ru—C4—C5	−129.1 (3)	C4—Ru—N2—C22	87.0 (13)
C2—Ru—C4—C5	−99.9 (2)	C6—Ru—N2—C22	17.1 (14)
C6—Ru—C4—C5	−27.7 (2)	C1—Ru—N2—C22	−6.0 (13)
C1—Ru—C4—C5	−64.2 (2)	Cl—Ru—N2—C22	−91.7 (13)
Cl—Ru—C4—C5	−18.4 (6)	C20—N2—C22—C21	−61.0 (15)
N2—Ru—C4—C8	54.0 (4)	C22A—N2—C22—C21	58 (11)
N1—Ru—C4—C8	−24.9 (4)	Ru—N2—C22—C21	122.9 (8)
C3—Ru—C4—C8	118.4 (5)	C20—N2—C22—C23	64.4 (15)
C5—Ru—C4—C8	−112.5 (5)	C22A—N2—C22—C23	−176 (11)
C2—Ru—C4—C8	147.6 (4)	Ru—N2—C22—C23	−111.7 (9)
C6—Ru—C4—C8	−140.2 (4)	N2—C22—C23—C24	172.0 (12)
C1—Ru—C4—C8	−176.6 (4)	C21—C22—C23—C24	−66.5 (13)
Cl—Ru—C4—C8	−130.9 (4)	C20—N2—C22A—C23A	−65 (2)
C3—C4—C5—C6	−0.4 (5)	C22—N2—C22A—C23A	−130 (10)
C8—C4—C5—C6	179.8 (3)	Ru—N2—C22A—C23A	109.6 (13)
Ru—C4—C5—C6	54.1 (3)	C20—N2—C22A—C21A	61 (2)
C3—C4—C5—Ru	−54.5 (3)	C22—N2—C22A—C21A	−4(9)
C8—C4—C5—Ru	125.7 (3)	Ru—N2—C22A—C21A	−124.4 (12)
N2—Ru—C5—C6	−156.3 (2)	N2—C22A—C23A—C24A	161.9 (17)
N1—Ru—C5—C6	128.8 (2)	C21A—C22A—C23A—C24A	39.6 (19)
C3—Ru—C5—C6	−103.2 (3)		
